# Differences in SARS-CoV-2 Vaccine Response Dynamics Between Class-I- and Class-II-Specific T-Cell Receptors in Inflammatory Bowel Disease

**DOI:** 10.3389/fimmu.2022.880190

**Published:** 2022-04-08

**Authors:** Alexander M. Xu, Dalin Li, Joseph E. Ebinger, Emebet Mengesha, Rebecca Elyanow, Rachel M. Gittelman, Heidi Chapman, Sandy Joung, Gregory J. Botwin, Valeriya Pozdnyakova, Philip Debbas, Angela Mujukian, John C. Prostko, Edwin C. Frias, James L. Stewart, Arash A. Horizon, Noah Merin, Kimia Sobhani, Jane C. Figueiredo, Susan Cheng, Ian M. Kaplan, Dermot P. B. McGovern, Akil Merchant, Gil Y. Melmed, Jonathan Braun

**Affiliations:** ^1^Cedars Sinai Cancer and Department of Medicine, Cedars-Sinai Medical Center, Los Angeles, CA, United States; ^2^F. Widjaja Foundation Inflammatory Bowel and Immunobiology Research Institute, Cedars-Sinai Medical Center, Los Angeles, CA, United States; ^3^Department of Cardiology, Smidt Heart Institute, Cedars-Sinai Medical Center, Los Angeles, CA, United States; ^4^Adaptive Biotechnologies, Seattle, WA, United States; ^5^Applied Research and Technology, Abbott Diagnostics, Abbott Park, IL, United States; ^6^Center for Rheumatology Medical Group, Los Angeles, CA, United States; ^7^Department of Pathology and Laboratory Medicine, Cedars-Sinai Medical Center, Los Angeles, CA, United States

**Keywords:** SARS-CoV-2 (COVID-19), mRNA vaccine, T-cell repertoire, inflammatory bowel disease, immunodeficiency

## Abstract

T-cells specifically bind antigens to induce adaptive immune responses using highly specific molecular recognition, and a diverse T-cell repertoire with expansion of antigen-specific clones can indicate robust immune responses after infection or vaccination. For patients with inflammatory bowel disease (IBD), a spectrum of chronic intestinal inflammatory diseases usually requiring immunomodulatory treatment, the T-cell response has not been well characterized. Understanding the patient factors that result in strong vaccination responses is critical to guiding vaccination schedules and identifying mechanisms of T-cell responses in IBD and other immune-mediated conditions. Here we used T-cell receptor sequencing to show that T-cell responses in an IBD cohort were influenced by demographic and immune factors, relative to a control cohort of health care workers (HCWs). Subjects were sampled at the time of SARS-CoV-2 vaccination, and longitudinally afterwards; TCR Vβ gene repertoires were sequenced and analyzed for COVID-19-specific clones. We observed significant differences in the overall strength of the T-cell response by age and vaccine type. We further stratified the T-cell response into Class-I- and Class-II-specific responses, showing that Ad26.COV2.S vector vaccine induced Class-I-biased T-cell responses, whereas mRNA vaccine types led to different responses, with mRNA-1273 vaccine inducing a more Class-I-deficient T-cell response compared to BNT162b2. Finally, we showed that these T-cell patterns were consistent with antibody levels from the same patients. Our results account for the surprising success of vaccination in nominally immuno-compromised IBD patients, while suggesting that a subset of IBD patients prone to deficiencies in T-cell response may warrant enhanced booster protocols.

## Introduction

Coronavirus-19 (COVID-19) infection is characterized by immune dysregulation (1–8), especially in elderly or immunocompromised patients with comorbidities (9, 10). Vector-based vaccines (i.e. Ad26.COV2.S by Janssen/Johnson&Johnson) and mRNA vaccines (BNT162b2 by Pfizer/BioNTech, mRNA-1273 by Moderna/NIH) (11, 12) are both in wide use in the United States, although there may be some advantages of the latter in safety, pharmacokinetics, and manufacturing (13, 14). Vaccine-induced immune responses include both humoral (antibody) and cellular (T-cell) immunity. While post-vaccination antibody responses in various immune-compromised populations have been described (10, 15, 16), T-cell responses have not been well-characterized. Understanding the mechanisms of the immune system’s response to COVID-19 vaccines is essential to understanding infection risk in vaccinated vulnerable populations including those with immune-mediated conditions.

A critical element of the immune response to both SARS-CoV-2 infection and vaccination is the T-cell compartment (7, 17–19). T-cells are essential for specific recognition of virus antigens, which occurs *via* T-cell receptor (TCR) binding *via* either MHC Class-I or Class-II antigen presentation (20, 21). MHC Class-I presentation signals CD8+ T cells, while MHC Class-II presentation signals CD4+ T-cells which mediate both inflammatory effector processes and antigen-specific antibody generation (22). Flow cytometry-based and ELISpot methods permit enumeration of CD4+ and CD8+ T-cell vaccine responses, but do not permit dissection of the clonal dynamics of the T-cell response (23–26). Peptide stimulation reveals antigen-specific populations which can be functionally assayed (27, 28), but individual TCR clones must still be sequenced. High-throughput next-generation sequencing has made TCR sequencing widely available (29, 30), and TCR repertoire profiles have been described following COVID-19 infection (31, 32). Antigen-specificity and Class-I/II-specificity are then derived by computational analysis of TCR repertoire sequences and *in vitro* validation (33, 34).

Inflammatory bowel disease (IBD) is characterized by an aberrant host immune response to commensal gut bacteria, and is often treated with immune-modifying therapies including thiopurines, corticosteroids, monoclonal antibodies targeting tumor necrosis (TNF)-α, integrins, and interleukin (IL)-12/23, and small-molecule inhibitors of janus kinase (JAK) (35). While immunocompromised populations are generally at increased risk for COVID-related complications, those with IBD have shown COVID complication risks generally similar to the non-IBD population irrespective of biologic therapy or small molecule use (36). Furthermore, those with IBD have robust cellular responses (37) and very high rates of post-vaccination anti-spike seroconversion (16), while those treated with anti-TNF therapies or corticosteroids may have lower quantitative antibody levels (15). Thus, patients with IBD provide an ideal opportunity to study differential effects of these immune-modifying therapies on immune responses following vaccination against SARS-CoV-2.

In this study, we utilized two large, diverse cohorts of patients to track the progression of mRNA vaccine response in the T-cell compartment.

## Methods

### Patient Cohort and Sample Collection

We studied IBD (16) and non-IBD HCW subjects (38) (n=521 patients total, **Table 1**), enrolled in an IRB-approved prospective registry at Cedars-Sinai between January and June 2021 (9, 10). For IBD patients, samples were collected longitudinally at the time of SARS-CoV-2 vaccine dose 1, dose 2 (when available), and 2 and 8 weeks after dose 2 (after dose 1 for vector vaccine participants) when possible. For HCW subjects, samples were collected at dose 1, and 8 weeks after dose 2. HCW subjects for this study were chosen from the available HCW registry by matching for the IBD age distribution. We quantified spike-specific and nucleocapsid SARS-CoV-2 antibody levels using the SARS-CoV-2 IgG-II assay (Abbott Labs, Abbott Park, IL). Self-reported COVID-19 were excluded from analyses except where specifically indicated.

**Table 1:** Study Cohort.

	IBD	HCW
n	297	224
race, n(%)		
Asian	7(2.36)	77(34.38)
Black or African American	4(1.35)	0
Multiple	4(1.35)	0
Other	15(5.05)	0
Prefer not to answer	3(1.01)	0
White	264(88.89)	147(65.63)
		
Hispanic, n(%)	15(5.05)	14(6.25)
		
Gender, female n(%)	166(55.89)	159(70.98)
		
Vaccine type, n(%)		
BNT162b2	154(51.85)	223(99.55)
Ad26.COV2.S	15(5.05)	–
mRNA-1273	128(43.10)	1(0.45)
		
Prior COVID-19 History, n(%)	15(5.05)	12(4.46)
		
Treatments, n(%)		
No Immune suppression	49 (16.50)	–
Anti-TNF	103(34.68)	–
Other biologics (anti-IL23, anti-integrin)	125(42.09)	–
Immunomodulators	49(16.50)	–
		
Age group, n(%)		
<30	22(7.41)	20(8.93)
30-40	86(28.96)	72(32.14)
40-50	67(22.56)	53(23.66)
50-60	52(17.15)	27(12.05)
60-70	41(13.8)	36(16.07)
70+	29(9.76)	16(7.14)

### Immunosequencing

Immunosequencing of the CDR3 regions of human TCRβ chains was performed on blood genomic DNA using the immunoSEQ Assay (Adaptive Biotechnologies, Seattle, WA), which includes bias-controlled multiplex PCR, high-throughput sequencing, identification and quantitation of absolute abundance of unique TCRβ CDR3 regions, and quantitation of the corresponding T cell fractions by template count normalization (33). Attribution of TCR sequences to SARS-CoV-2 spike or other non-spike SARS-CoV-2 protein specificities were assigned as described by Alter et al. and Snyder et al. (32, 39). Briefly, SARS-CoV-2-associated TCRβ sequences were identified using a one-tailed Fisher’s exact test comparing TCRβ presence in SARS-CoV-2 PCR-positive samples (n=1954) with negative controls (n=3903). Subsets of these SARS-CoV-2-associated sequences were assigned to spike and non-spike antigens based on data from multiplexed antigen stimulation assays (40). A total of 917 TCRs were assigned to the SARS-CoV-2 spike protein and 1564 to non-spike viral proteins. The breadth metric was calculated as the number of unique annotated rearrangements among total number of unique productive rearrangements in the individual sample’s dataset. The depth metric was calculated by the summation of two elements multiplied pairwise; (a) the raw frequency of each rearrangement in the total repertoire in the individual sample’s dataset, and an estimate of clonal generations of the lineage represented by each rearrangement. The resultant depth metric estimates the relative number of clonal expansion generations across the TCRs, normalized by the total number of TCRs sequenced in the sample. Hence, the metric can range from negative to positive values. We infer whether a SARS-CoV-2-associated sequence is a CD4+ or CD8+ T cell by statistically associating each sequence to a Class II or Class I HLA. HLA associations are derived from a set of 657 SARS-CoV-2 positive individuals who have genotyped HLAs. We built a binary logistic regression classifier with L1 regularization to determine which HLA best predicts the observed distribution of a given SARS-CoV-2-associated sequence across all HLA-typed cases. The L1 regularization strength was tuned to yield a single non-zero coefficient, giving a single inferred HLA association for each enhanced sequence. The inferred HLA associations are validated against the subset of SARS-CoV-2-associated sequence which overlap with our multiplex antigen stimulation assays.

### Analysis and Statistics

All data was analyzed using R version 4.1.2. The MHC Class-I/II residual score was measured by calculating the linear regression of Class-I and Class-II breadth with no restrictions on the y-intercept. To calculate the scaled and normalized z-score, the mean of the residuals was subtracted from each residual and divided by the standard deviation. The residual and z-scores describe how far each patient’s response lies from the regression in an absolute manner, rather than a proportional manner (i.e. fold change), which limits Class-I/II variation noise from patients with low breadth and depth metrics. Patients with z-score >1 or <-1 were denoted as extreme responders and either Class-I biased or Class-I deficient, respectively.

Statistics were performed using t-tests, generalized linear models, ANOVA and Tukey’s test, and chi-squared tests as indicated. Generalized linear models were used unless otherwise noted in the text. Comparison of TCR breadth and depth used a Mixed Linear Model across time points and Generalized Linear Model within time points. Where possible, inverse normal transformation was performed, and age and sex were included as covariates. Confidence intervals for binomial probabilities were computed using exact methods. Other analyses are specified in the individual figures. Analyses were restricted to individuals with mRNA vaccines and no prior COVID-19 experience unless stated otherwise. Figure symbols and p-values serve as a visual guide, exact p-values are described in the text.

## Results

### Clonal Breadth and Depth of IBD and HCW Participants

The demographics of the study cohorts are shown in **Table 1**. Both IBD and HCW cohorts were disproportionately female (IBD, 56%; HCW, 71%). Participant ages ranged from 19 to 83 years. Participants were grouped by decades of age into 6 groups, with the largest group being 30-39 years (n=158). By study design, the age distributions of the HCW subjects were matched to the IBD subjects. The IBD and HCW groups were similar in Hispanic/Latino ethnicity (~5-6%), but differed by race with a higher proportion of Asian subjects among HCWs (**Table 1**). All HCWs received BNT162b2 except for 1 subject who received mRNA-1273, while those with IBD received all 3 vaccines (n=154 BNT162b2, n=128 mRNA-1273, n=15 Ad26.COV2.S). IBD patients had either Crohn’s disease (CD, 211) or Ulcerative Colitis/Indeterminant Colitis (UC/IC, 93).

**Figure 1:**
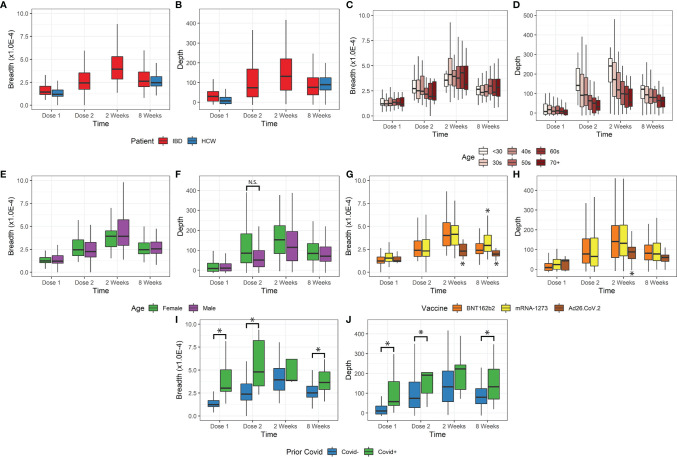
Breadth and depth metrics after COVID-19 vaccination. **(A, B)** Breadth and depth increases over time, peaking at 2 weeks after vaccine dose 2 in IBD. This increase was observed for both IBD and HCW cohorts. **(C, D)** Depth, but not breadth, was dependent on age. Younger patients have greater depth and more clonal expansion, with a similar number of unique clones detected. **(E, F)** Female patients only exhibited increased depth at dose 2 but were also younger. **(G, H)** Ad26.COV2.S vaccination resulted in lower depth. mRNA-1273 vaccination resulted in slightly higher breadth only at 8 weeks after dose 2. **(I, J)** Prior covid infection resulted in higher breadth and depth, except at 2 weeks after vaccination (*p<0.05). N.S., not-significant

The time course of the T-cell response is shown in **Figures 1A, B** for IBD and HCW. These trends were observed in IBD patients and the HCW control population (after adjustment for age, sex, and IBD type). Both TCR breadth and depth increased significantly over time after vaccination compared to pre-vaccination (**Figures 1A, B**). For the IBD cohort, the fold-responses for clonal depth at dose 2, 2 weeks post-dose2, and 8 weeks post-dose 2, compared to dose 1, were 1.8, 2.7, and 1.9 fold (p<1E-10 each). Similarly, clonal breadth increased over time relative to baseline (p<1E-10). In the HCW cohort, the response was also elevated at the one evaluated time post-vaccine (8 weeks post-dose 2, p<1E-10). There was no significant difference by race or between IBD types and HCW cohorts for either depth or breadth metrics (**Figures S1A, B**).

Age was a significant factor in the T-cell response (**Figures 1C, D**), but this effect was restricted to clonal depth (p=5.8E-9), not clonal breadth (p=0.63). The impact of age on clonal depth was not significantly different for IBD and HCW cohorts (ANOVA, p=0.07). Sex was not significantly associated with clonal breadth and depth overall (**Figures 1E, F**). However, we observed variations over time that may suggest that sex affects the dynamics of the T-cell clonal response. A sex difference was observed at Dose 2, with a higher depth in female IBD patients versus male patients, but this was conflated with a disparity in age distribution and hence not significant (p=0.13, IBD only, **Figures 1F**).

We also assessed the impact of vaccine type on the T-cell response (**Figures 1G, H**). Among the relatively small subgroup (N=15) receiving vector vaccine (Ad26.COV2.S), we observed a significantly lower clonal breadth (p=0.0034) and depth (p=0.015), at 2 weeks and 8 weeks post vaccination compared to mRNA vaccines. Responses to the two mRNA vaccines were generally comparable, but patients vaccinated with mRNA-1273 generated a higher clonal breadth (p=0.0044) but not depth response (p=0.53) in patients at 8 weeks.

Our data included a small number of subjects with self-reported prior COVID-19 infection (n=27). Despite a small sample size, this group was associated with significantly higher breadth and depth at dose 1, dose 2, and 8 weeks post vaccination (p=0.036E-7, **Figures 1I, J**). In accordance with the TCR response after COVID-19 infection, we also observed an elevated response non-spike, SARS-CoV2-specific T breadth and depth in subjects with prior infection. These cells are not stimulated by the vaccine and therefore unchanged following vaccination in subjects with no prior COVID-19 infection (p<1E-10, **Figure S1C**). Among the top 10% of non-spike breadth values, 27% of samples reported prior COVID-19 infection (26/95) compared to 2.1% (19/875) prior infections in the bottom 90% of non-spike breadth values. Similarly, of the top 10% of non-spike depth values, 20% (19/97) came from reported prior infection samples compared to 3% of the bottom 90% of non-spike depth values.

### Interplay and Dynamics Between MHC Class-I- and Class-II-Specific TCR Response

**Figure 2:**
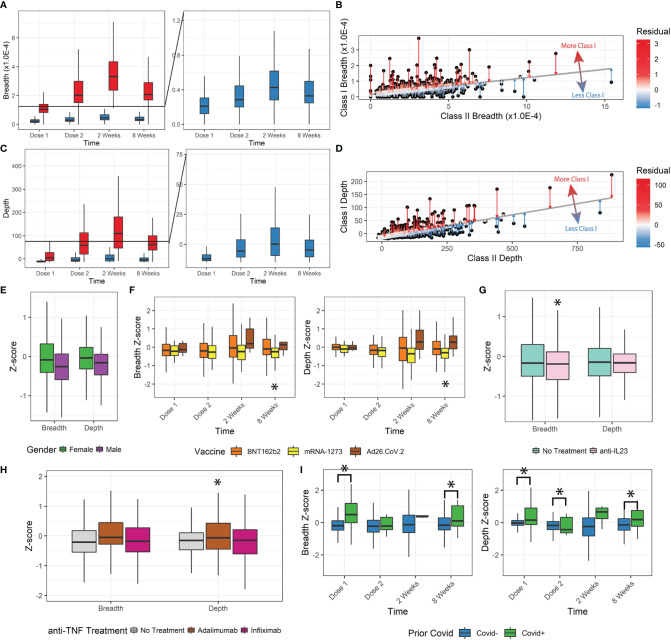
Class-I and Class-II TCR metrics. **(A, B)** Class-I-specific breadth was consistently lower than Class-II-specific breadth. The residual metric was used to describe how much Class-I-specific breadth was observed relative to expected. **(C, D)** The Class-I/II residual was replicated for depth. **(E)** Z-scores for female patients were higher. **(F)** Z-scores for mRNA-1273 patients were significantly lower. Ad26.COV2.S differences were not statistically significant with the small sample size, but trended higher. **(G, H)** Anti-TNF treatment by adalimumab but not infliximab increased depth z-scores, and anti-IL23 decreased breadth z-scores. **(I)** Prior covid infection was significantly associated with higher z-scores (*p<0.05).

We further analyzed the change in T-cell response by stratifying patients by the MHC Class-I/Class-II MHC-specific response. As stand-alone metrics, the Class-I- and Class-II-specific clonal breadth and depth responses display the same trends as the overall breadth and depth (**Figures 2A, C**). There was a consistent proportion of Class-I- to Class-II-specific clones for both breadth and depth (about 85% Class-II).

We tested whether there was any disproportionate distribution of Class-I/Class-II variation (from Class-I-biased to Class-I-deficient/relatively Class-II-biased TCR responses) that might be dependent on clinical factors. To measure the Class-I/II variation, we performed a linear regression between Class-I and Class-II breadth and depth and measured the residual (**Figures 2B, D**) and z-score of Class-I/II bias for each patient’s SARS-CoV-2-specific response (**Figures S2A, B**).

Lower z-scores indicate less Class-I-specific T-cell activity. We observed a small but significant decrease in z-score for breadth and depth with increasing age group (**Figures S2C-F**, ANOVA, breadth p=1.7E-8, depth p=1.8E-7). Z-score and thus Class-I-specific T cells increased in female participants (breadth p=0.003, depth p=0.006, **Figure 2E**). No significant changes were associated with the vaccination time (breadth p=0.44, depth p=0.24) or IBD status (breadth p=0.75, depth p=0.68). We also observed a divergence in breadth and depth z-scores over time by vaccine administered, with those that received mRNA-1273 having significantly lower z-scores (8 weeks breadth p=0.02, depth p=0.0035, **Figure 2F**).

Some immunomodulatory treatments significantly affected the Class-I/II balance. Patients receiving anti-IL23 (n=86, **Figure 2G**) had lower breadth z-scores (p=0.039) but not depth z-scores (p=0.11). No significant changes were observed in breadth or depth z-scores for patients receiving anti-integrins (n=40, breadth p=0.33, depth p=0.058) or immunomodulators (n=49, breadth=0.86, depth=0.26). Different types of anti-TNF treatment resulted in significant differences (n=103, infliximab n=63 and adalimumab n=37). After correcting for age, sex, and time after vaccination, patients receiving adalimumab had significantly higher depth Z-scores (p=0.012, **Figure 2H**) but not breadth Z-scores (p=0.091), while those receiving infliximab (breadth p=0.83, depth p=0.44) did not. Finally, patients reporting prior COVID-19 infections also had significantly higher z-scores and Class-I bias (p<0.05), especially at Dose 1 and at 8 weeks after vaccination (**Figure 2I**).

### Patterns in MHC Class-I and Class-II Extreme Responders

**Figure 3:**
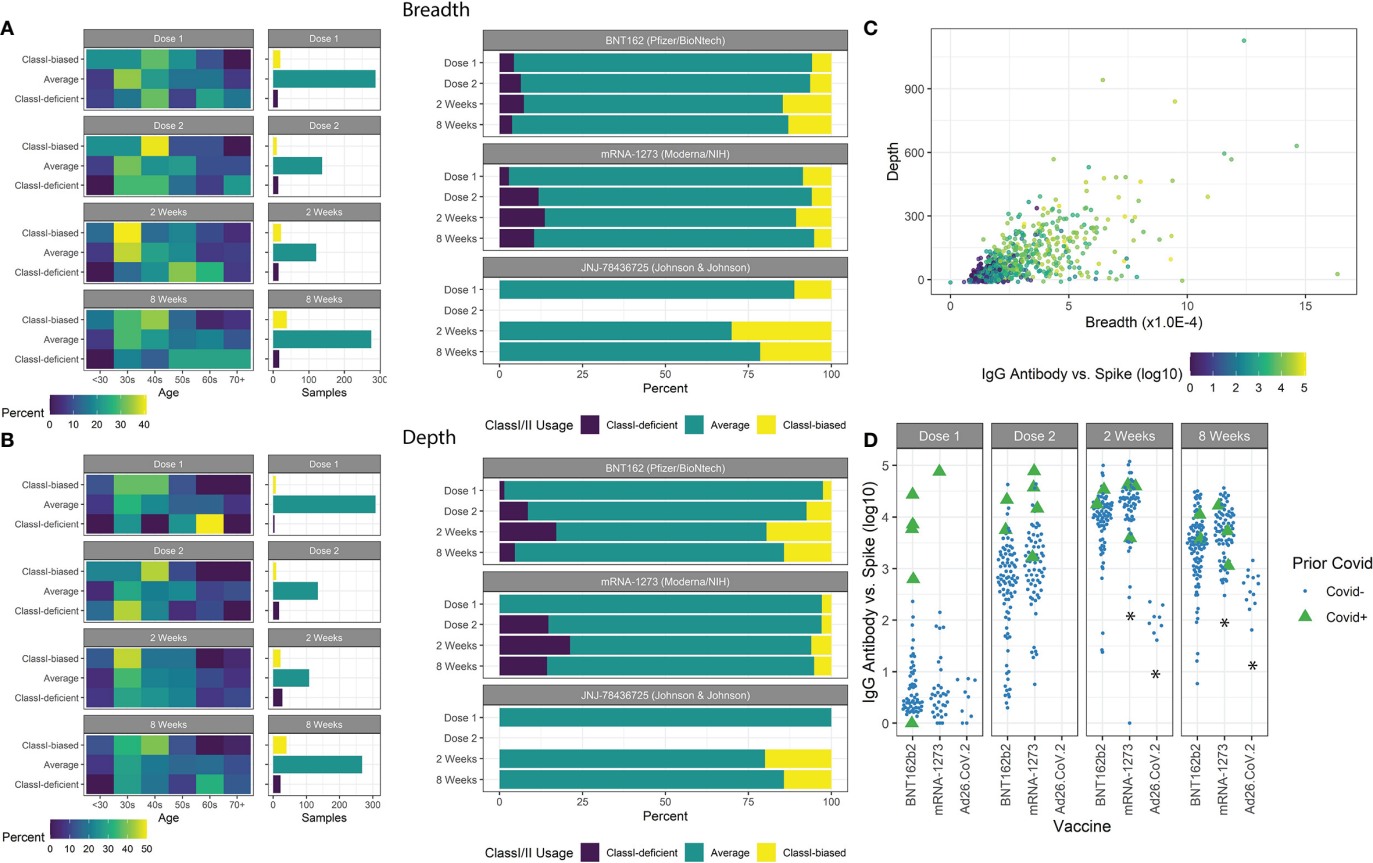
Class-I/II patient extremes. **(A)** Age groups of patients with breadth z-scores <-1, >1, and between -1 and 1 are shown with the number of samples in each group. Class-I-biased patients were more likely to be younger, while Class-I-deficient patients were more likely to be older. Ad26.COV2.S vaccines resulted in a higher proportion of Class-I-biased patients. **(B)** Similar trends were observed using depth z-scores. **(C)** Spike-specific antibody serology was correlated with both breadth and depth. **(D)** Prior covid infection was strongly associated with antibody counts. mRNA-1273 vaccination resulted in higher antibody counts and Ad26.COV2.S vaccination resulted in lower counts (*p<0.05).

For ~85% of all patients measured, a typical balance between measured Class-I and Class-II breadth and depth was maintained with Class-I:Class-II at ~1:6 ratio, indicated by Class-I/II z-scores between -1 and 1. Other extreme responder patients were isolated (**Figures 3A, B**) and associated with clinical features. The most significant associations were observed at 8 weeks after vaccination, with both age and vaccine type emerging (chi-squared test, breadth age p=0.032 and vaccine type p=0.038, depth age p=0.0095 and vaccine type p=0.018). Patients with breadth or depth z-scores greater than 1 (Class-I-biased) were significantly younger, and vice versa for z-scores less than -1 (Class-I-deficient). Of the three vaccines studied, patients receiving mRNA-1273 resulted in lower z-score extremes, while those receiving Ad26.COV2.S resulted in higher z-score extremes in the limited sample size.

We measured antibody abundance in patients to determine if TCR metrics were associated with antibody production (**Figure 3C**). As previously reported (16, 38, 41, 42), IgG-spike antibody levels were highly dependent on time after vaccination, prior SARS-CoV-2 infection, and vaccine type, and slightly dependent on IBD status (**Figure 3D**). No significant association was found based on age or gender (p=0.91, p=0.35, respectively). Antibody levels were highly correlated with standard TCR metrics of breadth and depth. We observed increased antibody levels with increased breadth z-score, independent of the overall clonal breadth (p=0.028).

## Discussion

The development of vaccines in response to the COVID-19 pandemic has been a successful public health initiative that has also revealed insights on human immunobiology. Among the present challenges are breakthrough infections and reduction in disease severity for emerging viral variants. The corresponding roles of antibody and cellular immunity are dependent on Class II and Class I T-cell vaccine responses, which are understudied compared to the vaccine-induced antibody response and sensitive to immunologic disease and immunotherapy. In this study, we assessed the antibody and T-cell vaccine responses, the latter distinguished by molecular TCR-based assessment of T-cell clonal dynamics, in a demographically diverse cohort of control and IBD patients. Our findings reveal preservation of the T-cell vaccine response across demographics and in IBD patients under a range of immune-targeted therapies. However, we observed impacts of subject age and vaccine type, affirming and expanding on recent reports of the T-cell response to vaccination (32, 39). This study adds to the growing body of evidence showing that immunocompromised status does not necessarily prevent a vaccination response (43, 44).

Compared to antibody serology, which in studies by our group and others is relatively constant in the initial post-vaccine period (16, 38), TCR metrics begin to diverge dependent on clinical features. Increased depth but not breadth in younger patients suggests that the elevated response of younger versus older patients was not *via* the recruitment of more novel antigen-specific clones, but instead by elevated burst size of the available T-cell clonal population (45). Higher depth was observed in patients vaccinated with mRNA-1273 at 8 weeks, suggesting more persistent retention of unique T-cell clones. Since vaccination elicits spike protein-specific TCR sequences, non-spike protein-specific sequences detect natural infection events. In patients with high (>90^th^ percentile) non-spike breadth or depth values, we observed a nearly 10-fold higher rate of prior COVID-19 infections (2% to 20%), but a majority of these patients still did not report a prior COVID-19 infection. These non-spike specific clones detected at high levels could be due to prior exposure to related viruses (46, 47), or a prior asymptomatic, unreported infection.

By measuring Class-I/II-specific TCR sequences, we explored distinct mechanisms of T-cell-mediated immunity. We initially hypothesized that a Class-I-biased response may be linked with relatively lower antibody levels due to proportionally less helper T-cell activation *via* Class-II-specific T cells. Perhaps surprisingly, a higher z-score breadth, which indicates Class-I-specific T-cell bias, was instead associated with increased antibody response. This phenotype most likely reflects a stronger comprehensive response, including both Class-I and Class-II-specific T cells. Indeed, no patients had a truly absent Class-II response while also having a Class-I response. Patients with prior COVID-19 infections also had a significant Class-I-biased response before vaccination, suggesting that Class-I-specific TCRs were the most significant holdovers of the immune response to natural infection. This bias disappeared at early stages of vaccination, but re-emerged after 8 weeks, suggesting possible differences in longevity to natural immunity and vaccine-induced responses.

These differences were more pronounced when considering extreme responders by Class-I/II variation. The vaccine type was a significant factor in the extreme response phenotype, including distinctions between the two mRNA vaccines analyzed in this study (48). No patients receiving Ad26.COV2.S displayed Class-I-deficient responses, and those receiving mRNA-1273 reporting ~3x the rate of Class-I-deficient responses as those receiving BNT162b2 (11% vs 4%). Due to the very low occurrence of breakthrough infection during the period of this study, we were unable to assess whether these extreme Class-I/II responses related to efficacy of protection.

The interpretation of this data has several caveats. First, a direct comparison of the absolute strength of the vaccine-induced response vs. the prior infection response was not possible, as the Adaptive Biotechnologies reference dataset was generated using natural infections, and vaccines might stimulate distinct TCRs (28). Moreover, matching of HLA-alleles will also affect the accuracy of TCR-based measurements, especially in minority populations. However, our analysis was illuminating in demonstrating that both vaccine-induced and natural infection-induced T cell clones were detectable in our study population. Second, these measurements were taken using the peripheral blood, which may not reflect the entire repertoire available, especially among Class-II-specific T cells participating in B-cell activity and maturation in lymph node compartments (49). Third, the z-score reflects a shift in balance between Class-I/II response, but this does not appear to preclude a patient with a Class-I-biased z-score from mounting an effective antibody response, which is instead correlated to the absolute magnitude of clonal breadth and depth. Fourth, individual TCR clones contributing to SARS-CoV-2-specific breadth and depth were not characterized for the magnitude of the cellular response by assays such as ELISpot or cytokine release. Finally, no COVID infections, symptomatic or asymptomatic, in our study subjects were reported during the window of observation, so it was not possible to assess the influence of TCR response or Class-I/II balance on disease prevention or severity.

TCR sequence-based analysis of antigen-specific responses and T-cell clonal dynamics quantifies an important element of adaptive immunity, but poses a formidable analytic challenge. This challenge can be addressed by the development of validated, large-scale clonal datasets of antigen-specific T cell responses, such as the SARS-CoV-2 dataset and associated metrics used in this study. These present findings uncover factors affecting the T-cell response that may guide SARS-CoV-2 vaccination decisions for IBD and =control patients.

## Data Availability Statement

The raw data supporting the conclusions of this article will be made available by the authors, without undue reservation.

## Ethics Statement

The studies involving human participants were reviewed and approved by Cedars-Sinai Institutional Review Board. The patients/participants provided their written informed consent to participate in this study.

## Author Contributions

AX, DL, JE, EM, RE, RG, HC, JP, EF, JS, AH, NM, GB, KS, IK, GM, and JB acquired data. All authors analyzed and interpreted data. AX and DL performed statistical analysis. AX wrote the first draft of the manuscript. AX and DL analyzed data. AX, JB, and GM edited the manuscript. All authors read, revised, and approved the manuscript. JE, JF, SC, DM, GM, and JB obtained funding. DM, AMe, GM, and JB supervised the study. AX, DL, DM, GM, and JB equally contributed to the study. All authors contributed to the article and approved the submitted version.

## Funding

This study was supported by the Leona M. and Harry B. Helmsley Charitable Trust, the Widjaja Foundation Inflammatory Bowel and Immunobiology Research Institute, and the National Institute of Diabetes and Digestive and Kidney Disease Grants P01DK046763 and U01DK062413, the Cedars-Sinai Precision Health Initiative, and the Erika J. Glazer Family Foundation. AX is supported by a Tower Cancer Research Foundation Career Development Award and NIH National Center for Advancing Translational Science (NCATS) UCLA CTSI Grant Number UL1TR001881.

## Conflict of Interest

GM has consulted for AbbVie, Arena Pharmaceuticals, Boehringer-Ingelheim, Bristol-Meyers Squibb/Celgene, Entasis, Janssen, Medtronic, Pfizer, Samsung Bioepis, Shionogi, Takeda, Techlab, and has received research funding from Pfizer for an unrelated investigator-initiated study. JB has received research funding from Janssen. DM has consulted for Takeda, Boehringer-Ingelheim, Palatin Technologies, Bridge Biotherapeutics, Pfizer, and Gilead, and is a consultant/stockholder for Prometheus Biosciences. RE, RG, HC, and IK are employees of Adaptive Biotechnologies. JP, EF, and JS are employees of Abbott.

The remaining authors declare that the research was conducted in the absence of any commercial or financial relationships that could be construed as a potential conflict of interest.

## Publisher’s Note

All claims expressed in this article are solely those of the authors and do not necessarily represent those of their affiliated organizations, or those of the publisher, the editors and the reviewers. Any product that may be evaluated in this article, or claim that may be made by its manufacturer, is not guaranteed or endorsed by the publisher.
