# Supplementary material for: Differences in SARS-CoV-2 Vaccine Response Dynamics Between Class-I- and Class-II-Specific T-Cell Receptors in Inflammatory Bowel Disease
Source: Front Immunol. 2022 Apr 8;13:880190. doi: 10.3389/fimmu.2022.880190 (PMC9024211; doi:10.3389/fimmu.2022.880190)
Supplement: Supplementary file 1 [file DataSheet_1.docx]

Differences in SARS-CoV-2 Vaccine Response Dynamics Between Class-I- and Class-II-Specific T-Cell Receptors in Inflammatory Bowel Disease

Alexander Xu, PhD^1^, Dalin Li, PhD^2^, Joseph E. Ebinger, MD, MS^3^, Emebet Mengesha, BS^2^, Rebecca Elyanow, PhD^4^, Rachel M. Gittelman, PhD^4^, Heidi Chapman, PhD^4^, Sandy Joung, MHDS^3^, Gregory J. Botwin, BS^2^, Valeriya Pozdnyakova, BS^2^, Philip Debbas, BS^2^, Angela Mujukian, M.D.^2^, John C. Prostko, MS^5^, Edwin C. Frias, MBA^5^, James L. Stewart, PhD^5^, Arash A Horizon, MD^6^, Noah Merin, MD, PhD^1^, Kimia Sobhani, PhD^7^, Jane C. Figueiredo, PhD^1^, Susan Cheng, MD, MMSc, MPH^3^, Ian M. Kaplan, PhD^4^, Dermot P.B. McGovern, MB BS, D Phil^2^, Akil Merchant, MD^1^, Gil Y. Melmed, MD, MS^2^, Jonathan Braun, MD, PhD^2,7^*

^1^Cedars Sinai Cancer and Department of Medicine, Cedars-Sinai Medical Center, Los Angeles, CA, USA

^2^F. Widjaja Foundation Inflammatory Bowel and Immunobiology Research Institute, Cedars-Sinai Medical Center, Los Angeles, CA, USA

^3^Department of Cardiology, Smidt Heart Institute, Cedars-Sinai Medical Center, Los Angeles, CA, USA

^4^Adaptive Biotechnologies, Seattle, WA, USA.

^~~5~~^Applied Research and Technology, Abbott Diagnostics, Abbott Park, IL5.

^6^Center for Rheumatology Medical Group, Los Angeles, CA, USA

^7^Department of Pathology and Laboratory Medicine, Cedars-Sinai Medical Center, Los Angeles, CA, USA

*** Correspondence:**Corresponding Author
Jonathan.braun2@csms.org


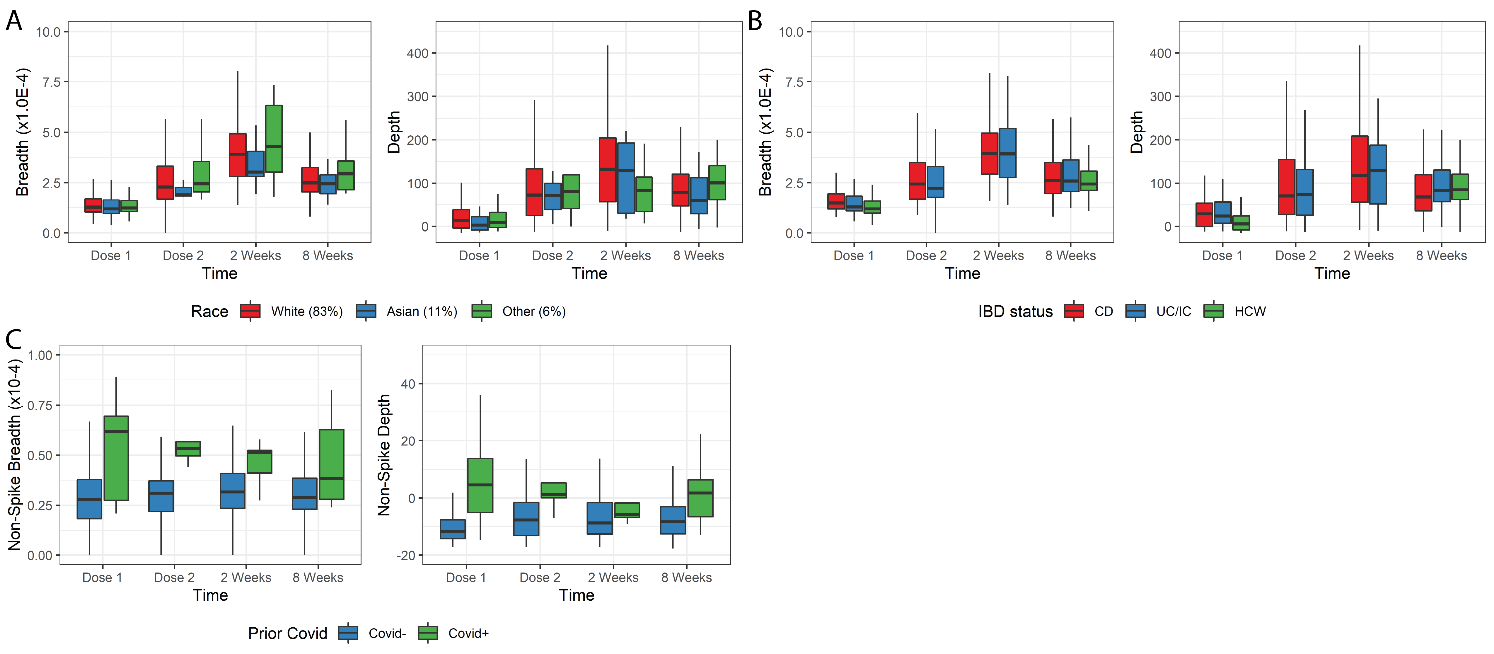


**Supplementary Figure 1.** Effect of race, IBD status, and COVID status on TCR response. A-B. Levels of T-cell response in relation to race and IBD status. C. Non-spike-specific breadth and depth dependence in COVID-experienced and COVID-naïve patients. Neither race nor IBD status significantly affected TCR breadth or depth. C. For patients reporting prior infection, TCRs specific to non-spike protein were significantly elevated, and the depth declined over time. For patients without prior infections, no change was observed over vaccination.


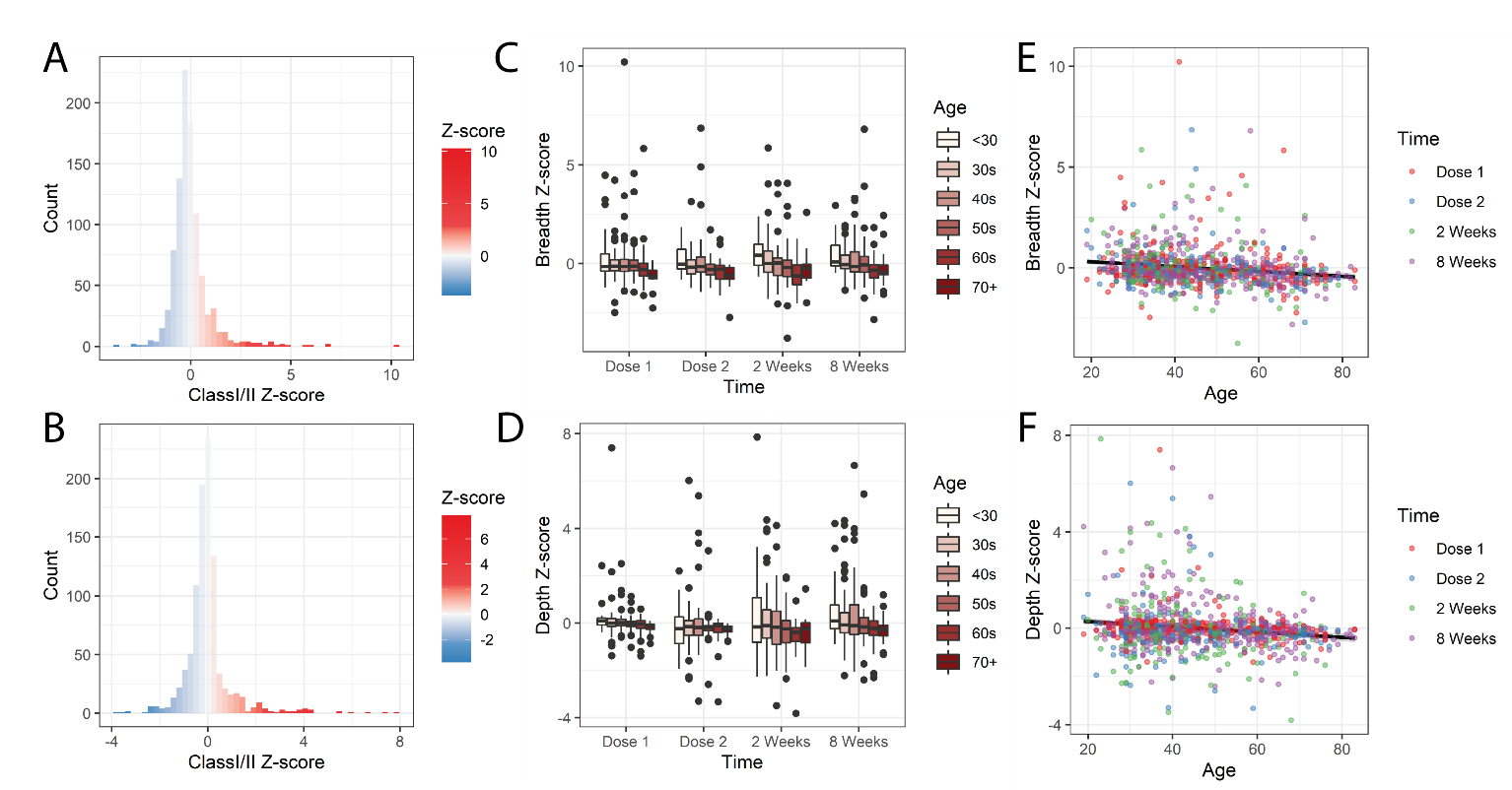


**Supplementary Figure 2.** Additional MHC Class I/II z-score metrics. A. Class I/II breadth z-score describes the normalized residual. B. Depth z-score is similarly distributed as breadth. C-D. Z-scores for breadth and depth decline with age groups, signifying relatively fewer Class I TCRs detected with increasing age. E-F. Z-scores decline with age, and no dependence on time of vaccination is observed.
